# PhenoApp: A mobile tool for plant phenotyping to record field and greenhouse observations

**DOI:** 10.12688/f1000research.74239.1

**Published:** 2022-01-07

**Authors:** Franco Röckel, Toni Schreiber, Danuta Schüler, Ulrike Braun, Ina Krukenberg, Florian Schwander, Andreas Peil, Christine Brandt, Evelin Willner, Daniel Gransow, Uwe Scholz, Steffen Kecke, Erika Maul, Matthias Lange, Reinhard Töpfer

**Affiliations:** 1 Julius Kühn Institute (JKI) - Federal Research Centre for Cultivated Plants, Institute for Grapevine Breeding Geilweilerhof, Siebeldingen, 76833, Germany; 2 Julius Kühn Institute (JKI) - Federal Research Centre for Cultivated Plants, Data Processing Department, Erwin-Baur-Straße 27, Quedlinburg, 06484, Germany; 3 Leibniz Institute of Plant Genetics and Crop Plant Research (IPK) Gatersleben, Corrensstraße 3, Seeland, 06466, Germany; 4 Julius Kühn Institute (JKI) - Federal Research Centre for Cultivated Plants, Data Processing Department, Königin-Luise-Strasse 19, Berlin, 14195, Germany; 5 Julius Kühn Institute (JKI) - Federal Research Centre for Cultivated Plants, Institute for Breeding Research on Fruit Crops, Pillnitzer Platz 3a, Dresden/Pillnitz, 01326, Germany; 6 Leibniz Institute of Plant Genetics and Crop Plant Research (IPK), The Satellite Collections North, Parkweg 3a, Sanitz, 18190, Germany; 7 Leibniz Institute of Plant Genetics and Crop Plant Research (IPK), The Satellite Collections North, Inselstraße 9, Malchow/Poel, 23999, Germany

**Keywords:** Android app, plant phenotyping, digital data acquisition, LIMS, BBCH, FAIR principles

## Abstract

With the ongoing cost decrease of genotyping and sequencing technologies, accurate and fast phenotyping remains the bottleneck in the utilizing of plant genetic resources for breeding and breeding research. Although cost-efficient high-throughput phenotyping platforms are emerging for specific traits and/or species, manual phenotyping is still widely used and is a time- and money-consuming step. Approaches that improve data recording, processing or handling are pivotal steps towards the efficient use of genetic resources and are demanded by the research community. Therefore, we developed PhenoApp, an open-source Android app for tablets and smartphones to facilitate the digital recording of phenotypical data in the field and in greenhouses. It is a versatile tool that offers the possibility to fully customize the descriptors/scales for any possible scenario, also in accordance with international information standards such as MIAPPE (Minimum Information About a Plant Phenotyping Experiment) and FAIR (Findable, Accessible, Interoperable, and Reusable) data principles. Furthermore, PhenoApp enables the use of pre-integrated ready-to-use BBCH (Biologische Bundesanstalt für Land- und Forstwirtschaft, Bundessortenamt und CHemische Industrie) scales for apple, cereals, grapevine, maize, potato, rapeseed and rice. Additional BBCH scales can easily be added. The simple and adaptable structure of input and output files enables an easy data handling by either spreadsheet software or even the integration in the workflow of laboratory information management systems (LIMS). PhenoApp is therefore a decisive contribution to increase efficiency of digital data acquisition in genebank management but also contributes to breeding and breeding research by accelerating the labour intensive and time-consuming acquisition of phenotyping data.

## Introduction

Classical breeding of agronomical improved crops relies on crossing of desired genotypes, growing their offspring and performing genotypic and phenotypic selection on the various traits. Desired genotypes consist of elite breeding material as well as genetic resources that are characterized. Approximately 7.4 million accessions are stored in around 1750 *ex-situ* germplasm collections (genebanks) worldwide. ^1^ These genebanks contain genetically and phenotypically diverse plant material and are excellent resources of novel traits useful for future plant breeding purposes in the context of changing demands.

Although innovative breeding technologies like marker-assisted selection have dramatically evolved to improve selection accuracy and intensity during recent decades, ^2^^,^^3^ only a small number of genotypes contributed directly to modern crop cultivars mainly due to the lack of sufficient phenotypic and genotypic characterization or limited evaluation of agronomic traits. ^4^^–^^6^ The efficient use of germplasm collections as a source of genetic variation is time consuming and arduous and therefore still remains a challenging task. Thus, with the development and decreasing costs of genotyping and sequencing technologies, genebank phenomics display the current bottleneck for the utilization of genetic resources. ^7^^–^^9^ Nevertheless, tremendous advancements in high-throughput phenotyping technologies during recent years are closing this gap. ^10^^–^^12^ However, as long as these high-throughput phenotyping technologies are a limiting factor, e.g. due to missing technologies, infrastructure or funding, supporting tools for manual data acquisition and recording can accelerate and support the evaluation of genetic resources and can increase breeding efficiency.

Plant phenomics is a multidisciplinary field developing novel sensing and imaging techniques for high-throughput phenotyping of plant genetic resources, and has applications in breeding. ^13^^–^^15^ The research basis are morphological, agronomical, physiological and metabolic features, whereas the handling, processing and adequate utilization of data is still challenging. The big advantage of high-throughput platforms compared to manual plant phenotyping is its objectivity as well as the time and cost-effectiveness. However, the development is slow and a plethora of research needs to be done to provide further valuable screening tools with practical use in the future. ^16^ With all these developments, the volume of data potentially usable for plant breeding has increased rapidly and will further increase in future. Therefore, the type of data generated range not only from agronomic and breeding-relevant phenotypic data, to results from quantitative and qualitative genetics, but can contain further information on fertilization, plant protection, field and soil conditions, geodata and weather data. Most data sets differ not only in their object of investigation, but also in their type, format and context of origin. The establishment of the FAIR principles (Findable, Accessible, Interoperable and Reusable) are therefore an important basis regarding harmonization and can increase the efficiency of plant breeding. ^17^^–^^19^ Another requirement is the interoperability of data in terms of machine-readable access to support continuous data analysis flows. To address the resulting challenges, internationally recognized work has already been done in the area of metadata and the minimum information standard for plant phenotyping data - MIAPPE - has been developed. ^20^ In addition, a large number of semantic resources exist, e.g. AGROVOC (word combination of agriculture and vocabulary) ^21^ or the general ontologies of the Open Biological and Biomedical Ontology (OBO) Foundry. ^22^ In the area of data structures, concepts for generalization have been proposed, such as Investigation-Study-Assay (ISA-TAB) ^23^ or, more recently, the Core Scientific Dataset Model, ^24^ which abstracts individual data structures to a self-describing generic data structure.

To ensure proper understanding of the content, we rely on common assessment and observation protocols, vocabularies and units. Many initiatives use their dedicated scoring scheme or, in the case of collaborative efforts such as genebanks, a commonly agreed set of observation protocols. ^25^ Prominent examples for scoring schemes are Darwin Core (DwC), ^26^ BBCH (Biologische Bundesanstalt für Land- und Forstwirtschaft, Bundessortenamt und CHemische Industrie) for phenology ^27^ and the International Union for the Protection of New Varieties of Plants ( UPOV) lists for morphological traits. The manual acquisition of such field phenotyping data into local infrastructures is a first component in a data publication pipeline.

A tool support for field phenotyping, i.e. observation scoring, enables the re-usability and fulfils the FAIR criteria. For this purpose, genebanks, research units, breeding companies or other stakeholder in plant phenotyping use not only simple, handwritten records, but also simple digital forms of recording. Microsoft Excel installations on mobile devices with or without voice input support, and solutions closely embedded in individual database infrastructures, such as the Genebank Information System/Bonitur (GBIS/B) at the Leibniz Institute of Plant Genetics and Crop Plant Research (IPK) ^28^ or recently published apps like vitisBerry, ^29^ SeedCounter ^30^ or Plant Screen mobile ^31^ may address specific demands, but lack convenience or generalization. As an alternative to such proprietary, hand crafted and/or non-generic solutions for recording and transferring field-scoring data into a digital representation or even databases such as Lab Information Management Systems (LIMS), an in-field digitalization of observations on a general basis represents the initial process to advance the field-data acquisition process.

In the current manuscript, we present an easy-to-use Android app (PhenoApp) for recording field and greenhouse observations on mobile devices to relieve the labor intensive and time-consuming process of manual phenotyping. All evaluated descriptors/scales can be individually created with maximum customization, allowing a fast and seamless data transfer into a digital representation for further utilization in genebank management and/or breeding research.

## Methods

### Implementation

***Data import and export***

**Figure 1.**
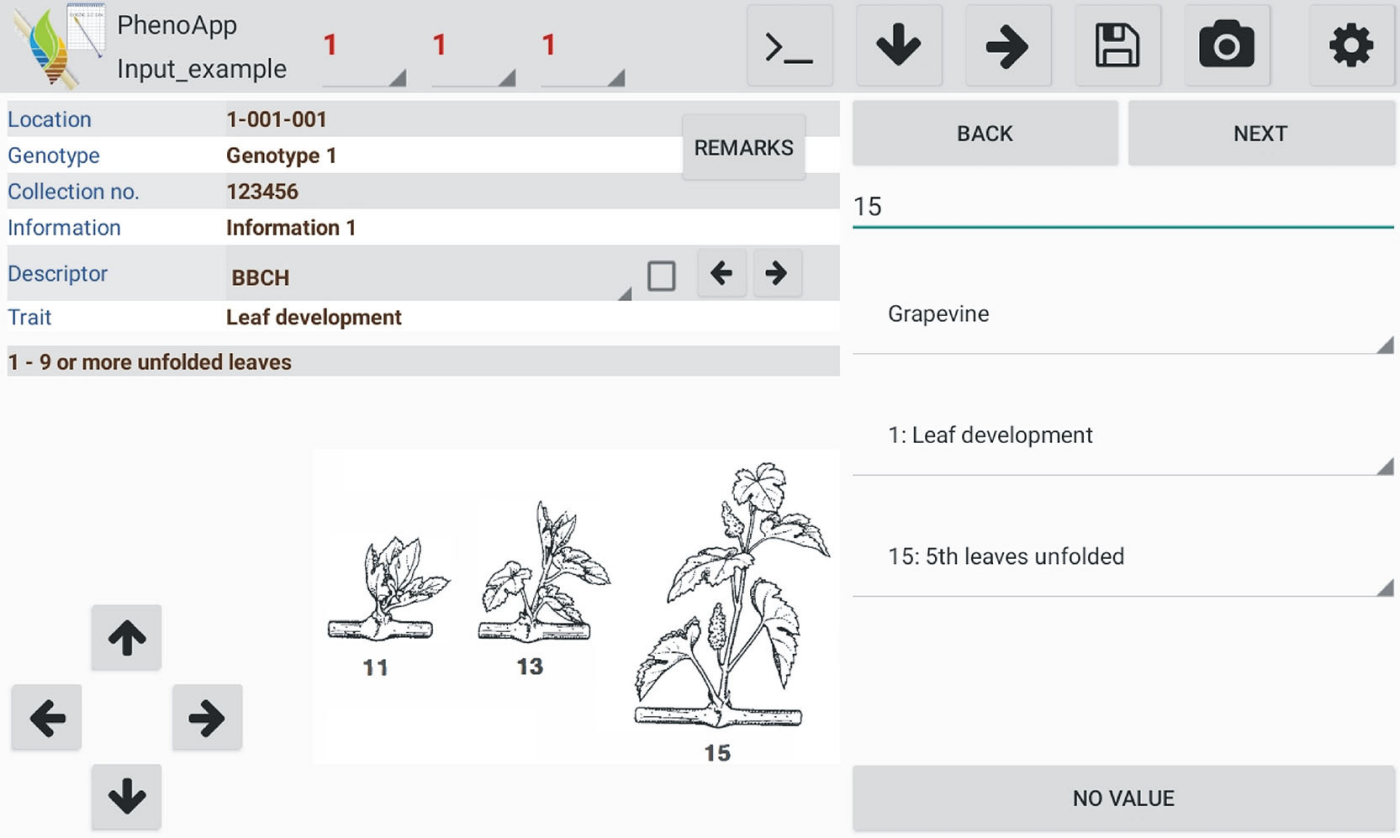
General app view with a data-recording example for a BBCH (Biologische Bundesanstalt für Land- und Forstwirtschaft, Bundessortenamt und CHemische Industrie) growth stage of grapevine.

After installation, the main directory contains an ‘in’ and ‘out’ folder. Input files need to be copied in the ‘in’ folder. Sample input files are then automatically displayed and selectable in the app entry page. The save button in the upper right corner of the app ( Figure 1) creates an output Excel file saved in the ‘out’ folder.

***Input file with descriptor list***

The first sheet of the input file (Locations) contains information on the specific plant locations with the columns plot, row, plant, accession name and number, variety number, genotype, mother, father, information and database-key. The last column (database-key) is not visible in the application and is used in the output file for better synchronization with the database. Only the first three columns or as alternative the fourth column with information on the exact plant locations are obligatory fields, all others are optional and implemented for better data handling and management as well as to give the user additional orientation and information.

The second sheet (Traits) contains all descriptors. These are described in the five columns which are shortcut (trait abbreviation), description, type, values and remark. The column type needs to be filled with 1 to 5 according to the format type of the recorded phenotypic data:

1. Rating: For the acquisition of categorical data. The scale needs to be specified in the value column (for example, 1: none; 3: little; 5: medium; 7: strong; 9: very strong).
2. Measured value: PhenoApp displays a numeric keypad for data entry.
3. Date: PhenoApp displays selectable buttons for today, yesterday, day before yesterday, tomorrow, day after tomorrow and a button for an individual date selection. Data are stored in numerical date format.
4. Text: PhenoApp opens a keyboard for free text input.
5. BBCH: PhenoApp displays the pre-integrated BBCH scales for phenotyping of phenology.

An example input file is provided in *Underlying data.* ^35^

***Output file***

The structure of the output file is the same as for the first sheet of the input file (specific plant locations) with additional columns for remarks and phenotyped data. Output format can be an Excel file or a csv (comma separated values) file for better machine readability. An example output file is provided in *Underlying data.* ^35^

***Important app functions***

*Phenotyping phenology based on BBCH scales*

As an additional mode for a convenient phenotyping of phenology over a certain time, PhenoApp provides pre-integrated BBCH scales without the need of own descriptors. Furthermore, if the option “BBCH question” is selected, each time PhenoApp is opened, it asks if the creation of a BBCH entry with the current date is desired. The integrated scales contain three list levels from species to principal growth stage to specific growth stage ( Figure 1). The release version has the BBCH scales of apple, cereals, grapevine, maize, potato, rapeseed and rice already implemented.

If needed, the user can implement scales of other crops or add own scales using an Excel template (bbch_template.xls) located in the ‘bbch’ folder of the app main directory (see *Software availability* ^36^). The first sheet is for the species as well as the principal growth stages and all further sheets for the specific growth stages. In addition, there is the option of adding sample images for each principal growth stage, which can be displayed in the app. To do this, the images need to be stored with the same filename as the stages in the app's ‘bbch’ folder together with the template file. The next time PhenoApp is started, the new BBCH scale will be imported to the internal database for further use and files are automatically deleted from the ‘bbch’ folder.

*Displaying sample images of descriptors*

If sample images of a descriptor with the same name as the descriptor shortcut are copied in the ‘descriptorPictures’ folder of the main directory, PhenoApp displays that image during phenotyping the same way as for BBCH scales.

*Taking pictures with the device camera*

The user can take one or several photos at any time. Clicking on the camera icon automatically opens the device standard photo app. Once a desired photo has been taken, it is saved in the subfolder ‘fotos’ in the app’s main directory. The file name of the photo is a combination of the currently specified location, the actual time stamp and the variety name (if given).

*Taking general and location specific notes*

Centered in the upper half of the screen is a ‘REMARKS’ button, which opens a window with two text fields for the input of specific location information and any additional information on the current plant, respectively. The text entries of both fields are included in the output file in separated columns. This feature gives the user the possibility to take additional notes during phenotyping without the need for further apps or handwritten remarks.

*Partial selection of descriptors*

It is possible to mark specific descriptors (square in the descriptor row, Figure 1) for selective data acquisition. Thus, the app only asks for indicated descriptors while all the others are skipped. This is particularly useful for phenotyping of multiple traits over time because it removes the necessity to manually skip descriptors not relevant at a specific time point.

*Arrow keys displayed in the lower left corner*

The arrow keys allow for a fast location jumping within a single row (up and down) or between rows (left and right).

*Language*

PhenoApp contains two language packages, English and German. The default language is English. If the global language setting of the mobile device is German, the language setting of the app changes automatically from English to German.

***Important app settings***

*Zigzag mode*

The app automatically jumps to the next listed descriptor after recording a data point, until the end of the descriptor list is reached. Then it moves directly to the first descriptor of the next location and so on. Logically, but quite unhandy in many practical cases, after reaching the end of a row, the app jumps to the first location in the next row (after the last row in a plot, it jumps to the next plot). To avoid walking unnecessary distances or the manual location correction, the app jumps in “zigzag” from the end of a row to the last location of the next row and counts backwards from there. This allows a continuous phenotyping while going through a field plot one row upwards and the next row backwards.

Arrow keys displayed in the top line give the direction of the next descriptor/location during phenotyping and are adaptable by a simple click on the button.

*First empty button*

This is a button on the top line that allows the user to jump to the first/next empty entry in their records. This is particularly useful for phenotyping multiple traits over time because it allows a fast tracking of missing data points.

*Accession, passport, genotype, parents, collection no. and information*

Displays the respective entry from the input file in the app if data are given.

*Left-handed mode*

The left-handed mode swaps the left and right side of the display for individual preferences.

*Multiple selection*

If the descriptor is of type ‘rating’, this option allows the user to select multiple values for a single trait. Results are separated with a comma in the output file. The activation of this setting removes the automatic jumping of the app to the next descriptor/location.

### Operation

PhenoApp was developed with Java 8 using Google’s appcompat-v7 and Apache’s POI libraries. It works on mobile phones and tablets with android API-level 14, which stands for android version 4.0 (Ice Cream Sandwich), or higher and requires no internet connection. The software is open source under Apache 2.0 license (see *Software availability* ^36^).

**Figure 2.**
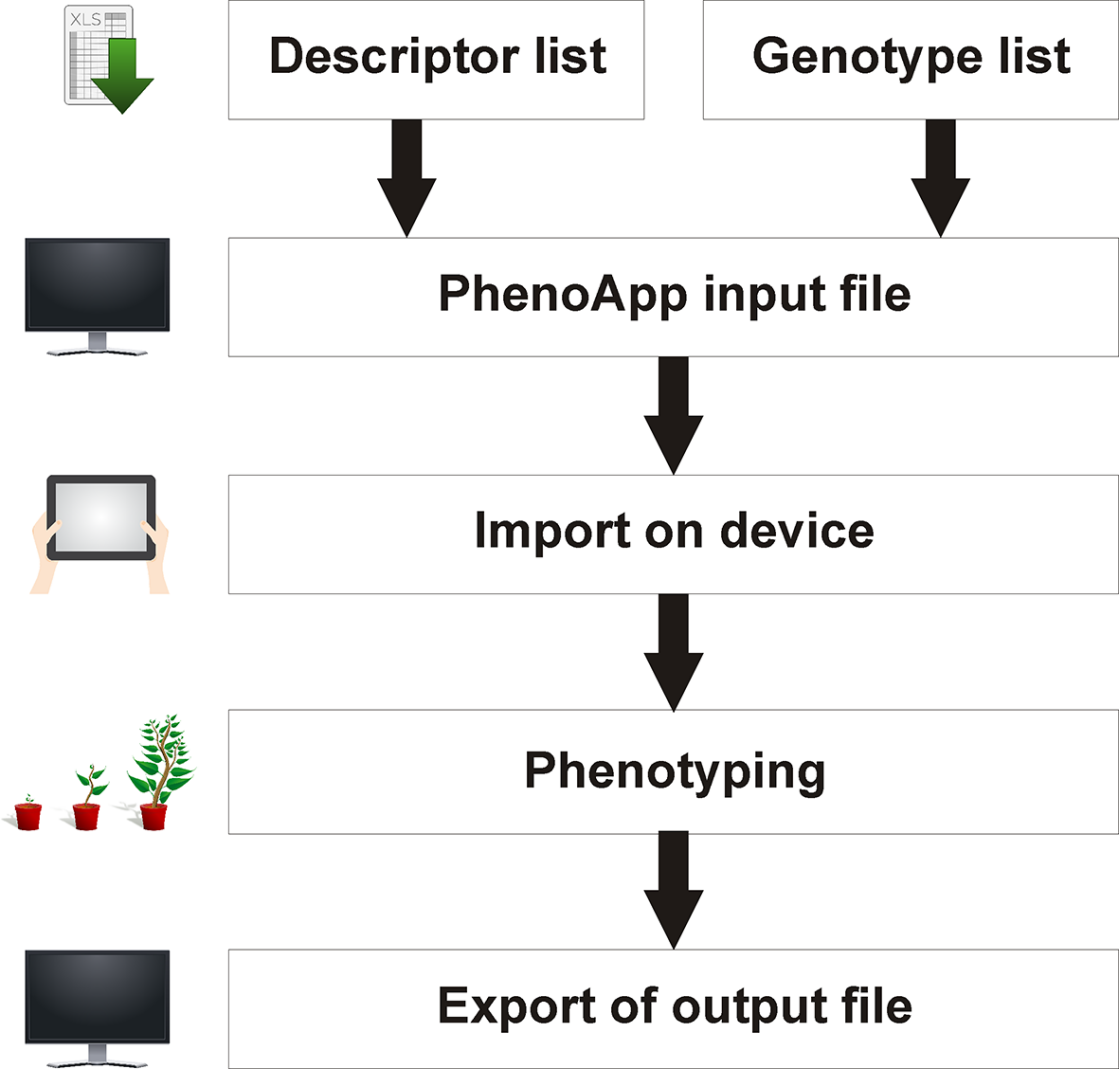
Principal PhenoApp workflow. A combined genotype-location and descriptor list is required as input. Post phenotyping, recorded data can be exported as a simple Microsoft Excel file. Minimal requirements are the PhenoApp on a mobile Android-based device and a computer with a spreadsheet software for data handling.

In general, PhenoApp is built as a simple interface to record phenotypic data. The app displays imported Microsoft Excel files containing a genotype and descriptor list and allows a data recording that can be saved and exported as Excel file for further use ( Figure 2).

## Use cases

### Default

For most cases, PhenoApp can/will be used to digitally support phenotypic data acquisition in the course of specific projects or routine work. It only needs a computer with Microsoft Excel and a plant list (incl. locations), a defined scientific question that can be translated into a descriptor, and a mobile Android-based device. The data integration in superordinate structures or the descriptor adaptation according FAIR principles are desirable and emphasized, but not initially required.

### Integration into local databases

For more than 10 years, IPK and the Julius Kühn Institute (JKI) have implemented a laboratory information management system (LIMS) as a universal platform for documenting and recording field, laboratory and bioinformatics data in order to establish a comprehensive system for research data documentation.

**Figure 3.**
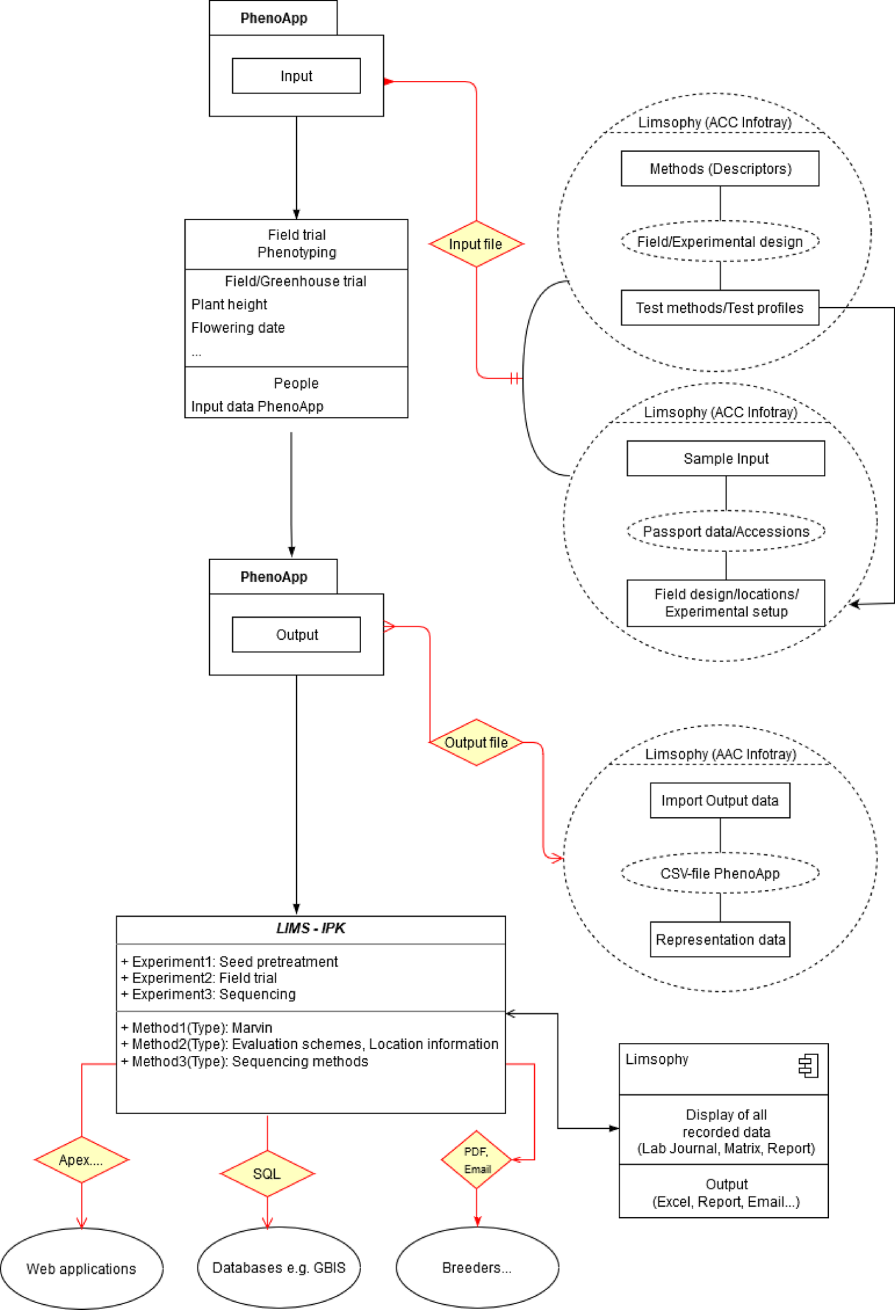
Data capture and integration into the laboratory information management system (LIMS) infrastructure of the Leibniz Institute of Plant Genetics and Crop Plant Research (IPK).

From this starting point, in a collaboration of IPK and JKI, PhenoApp was integrated in the LIMS workflow to advance data acquisition by allowing a fast, user friendly and direct import and export of datasets without the need of further modifications ( Figure 3). Using the well-defined, file based data exchange interfaces of PhenoApp, we embed the creation of necessary import files into the LIMS. Even a tight integration for an optimal data re-usability and interoperability was straightforward to implement, for example the storage and versioning of scoring methods. This, in turn, enabled mapping of scoring schemes for the same species across observation cycles and projects by linking various rating schemes with all scoring schema details and even reference images. In addition, a user-friendly input mask in the system provides the possibility of precisely documenting the locations of the corresponding plants. This enables the re-use and thus optimal interoperability across field phenotyping studies.

**Figure 4.**
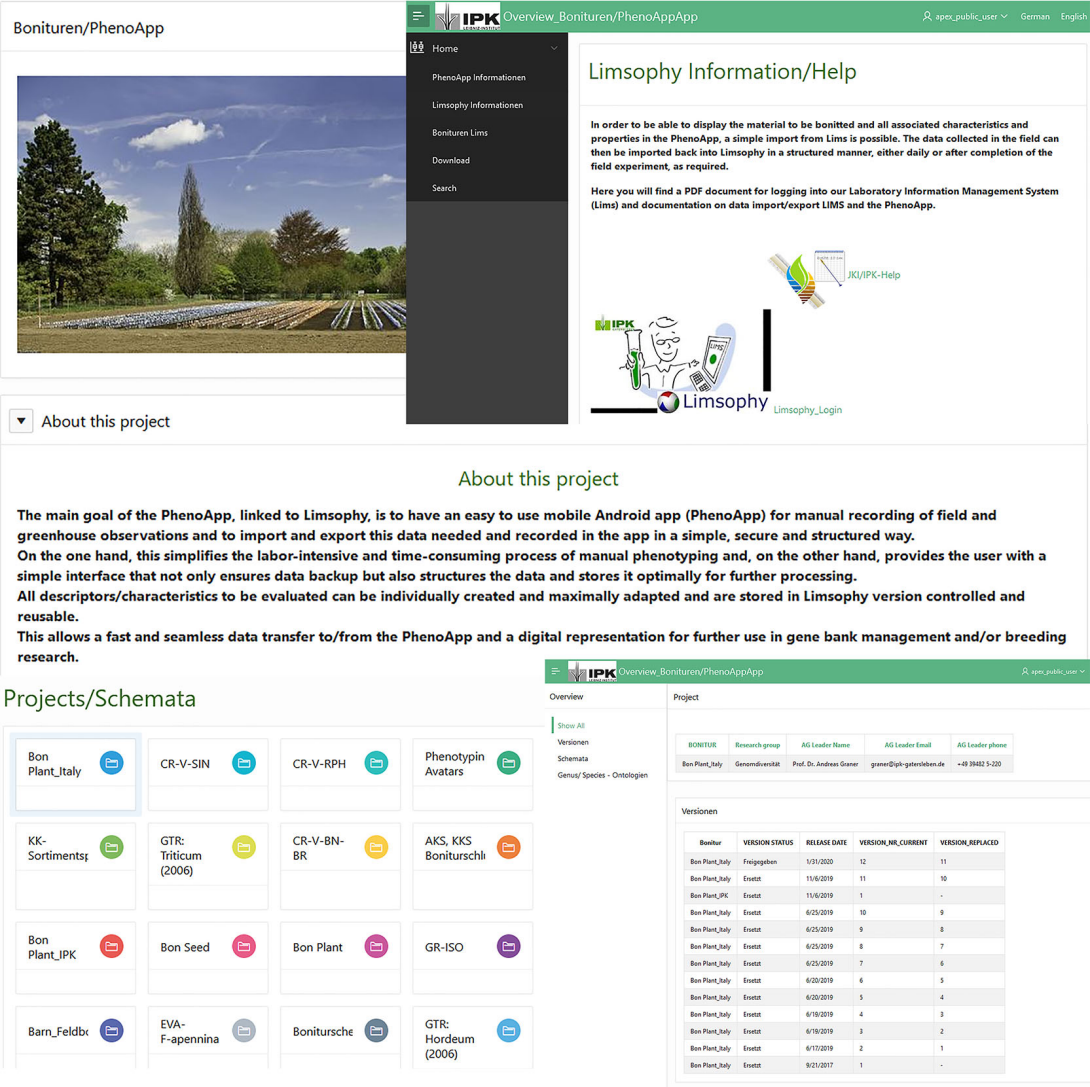
Overview of the Leibniz Institute of Plant Genetics and Crop Plant Research (IPK) intern Apex (Oracle Application Express) application. Here, users will find a compilation of the data already registered in the laboratory information management system (LIMS), i.e. given descriptors and scales of the genebank as well as additional information such as specific contact persons. This offers a wide range of applications and enables quick and clear access to existing data and information for the development of new experiments and routine work.

The data recorded by PhenoApp are exported as a well-structured CSV file and pictures taken stored into a specified folder. When a phenotyping run is finished, this enables an ad-hoc and consistent import into the LIMS database. The potential of such FAIR enabled field phenotyping is the pre-cursor to feed Web information systems ( Figure 4), data publication pipelines and APIs. ^32^ Currently, PhenoApp is used at the IPK within the barley pan-genome research project “ SHAPE II”. Based on a defined so-called core set, ^33^ representative barley genotypes were selected, grown in different years at IPK and scored with PhenoApp.

## Conclusions

PhenoApp provides a convenient and free-of-charge interface for a manual, digital and mobile data acquisition of a diverse range of phenotyping data. The user can easily create and customize their own descriptors and phenology scales. A simple entry to the application is possible for every user with basic knowledge of handling an Android device and spreadsheet software like Excel, without the need of integration in data management systems, although this is possible as demonstrated. Adaptations according to FAIR data principles ^17^ or international information standards like MIAPPE ^20^ or EURISCO ^34^ are possible without any restrictions. Furthermore, the resulting input and output files can easily be integrated in workflows of data management systems. The benefits can range from a facilitated project-specific phenotyping in the short term to a support during routine work in the medium term towards a complete harmonization of an institutional wide data infrastructure in the long term. In particular, datasets that have been uniformly collected and stored for years in genebanks or breeding facilities bear a huge potential to accelerate the efficient use of genetic resources and therefore the development of new adapted crop varieties that ensure future food security.

Thinking further, PhenoApp was initially developed for, but is not limited to plant phenotyping. The app is applicable whenever a manual data recording is required and a specific location is given. An input format of three separated parts (plot – row – individual) is required, but can easily be adapted or filled with placeholders to expand the application range.

## Data availability

### Underlying data

Zenodo: PhenoApp Input/Output files. https://doi.org/10.5281/zenodo.5760899. ^35^

This project contains the following underlying data:

- Input_example.xls (sample input file read in by PhenoApp. In addition, after installation, a sample input file will automatically be copied to the ‘in’ folder of the app main directory and no additional source data is required).
- Output_example.xls (sample output file created by PhenoApp).

Data are available under the terms of the Creative Commons Zero “No rights reserved” data waiver (CC0 1.0 Public domain dedication).

## Software availability

Source code available from: https://gitea.julius-kuehn.de/JKI/pheno-app

Archived source code at time of publication: https://doi.org/10.5281/zenodo.5525779 ^36^

License: Apache-2.0
