# Supplementary material for: PhenoApp: A mobile tool for plant phenotyping to record field and greenhouse observations
Source: F1000Res. 2022 Nov 28;11:12. Originally published 2022 Jan 7. [Version 2] doi: 10.12688/f1000research.74239.2 (PMC9813448; doi:10.12688/f1000research.74239.2)
Supplement: Peer review history

# Peer review: Reviewer response for version 2

**DOI:** 10.5256/f1000research.141539.r156817

**Published:** 2023-01-04

**Authors:** Andrew P. French

**Affiliations:** 1 University of Nottingham, Nottingham, UK

Thank you for the clear response to my comments - I am happy to recommend an approved rating for the article now.

Are the conclusions about the tool and its performance adequately supported by the findings presented in the article?

Yes

Is the rationale for developing the new software tool clearly explained?

Yes

Is the description of the software tool technically sound?

Yes

Are sufficient details of the code, methods and analysis (if applicable) provided to allow replication of the software development and its use by others?

Partly

Is sufficient information provided to allow interpretation of the expected output datasets and any results generated using the tool?

Yes

Reviewer Expertise:

Automated image analysis in plant phenotyping

I confirm that I have read this submission and believe that I have an appropriate level of expertise to confirm that it is of an acceptable scientific standard.

# Peer review: Reviewer response for version 2

**DOI:** 10.5256/f1000research.141539.r156818

**Published:** 2022-12-13

**Authors:** Karin Köhl

**Affiliations:** 1 Max Planck Institute of Molecular Plant Physiology, Potsdam, Germany

I thank the authors for their response and declare that I am satisfied with the information given. My only remaining request is that the recommendation to use a script to produce the input files is included in the conclusions.

Are the conclusions about the tool and its performance adequately supported by the findings presented in the article?

Partly

Is the rationale for developing the new software tool clearly explained?

Yes

Is the description of the software tool technically sound?

Partly

Are sufficient details of the code, methods and analysis (if applicable) provided to allow replication of the software development and its use by others?

Partly

Is sufficient information provided to allow interpretation of the expected output datasets and any results generated using the tool?

Partly

Reviewer Expertise:

NA

I confirm that I have read this submission and believe that I have an appropriate level of expertise to confirm that it is of an acceptable scientific standard.

# Peer review: Reviewer response for version 1

**DOI:** 10.5256/f1000research.77970.r140861

**Published:** 2022-07-29

**Authors:** Andrew P. French

**Affiliations:** 1 University of Nottingham, Nottingham, UK

Dear authors,

 This paper describes a potentially useful tool to address the issue of manual data collection from plant phenotyping experiments. Such a tool, if well executed, goes some way to providing for an information management system for collecting and recording phenotyping data in the field.

 I have more experience with automated data collection though analysis of images, but I do recognise the need and utility of a tool to assist with data capture by hand. 

 The app seems to focus almost entirely on data collection, and as noted in a response to a previous review, this data can then be fed to further downstream processing outside the app as required. The app therefore seems to be a semi-constrained front end for entering data into an Excel/CSV format. Whilst a simple idea, I believe this is useful in practice. I have some comments below.

 It is a shame that there is no enforcing of controlled vocabulary on some of the fields. In the previous response to authors it is noted that the use of internationally-recognised descriptors is recommended, so not having the option to enforce this in some scenarios feels like a missing feature which should be present.

 Related, a lot of emphasis is put on the input file, which I believe you use a lab information system to supply. How well practically do you see this working if the institute does not have a LIMS?

 I agree with the previous reviewer raising questions about Excel as a choice of data format. I think there are issues with this, and perhaps more flexible formats like JSON should be explored in the future (as you mention). However, there is no doubt Excel provides accessibility and readability of data in a form most phenotyping researchers will be familiar with.

 The option of uploading images is welcome, especially as I can see the benefit in later using these alongside the manually collected data to support development of AI-based systems which can analyse the images. However, only allowing one image at a time seems like an oversight here. I am convinced it would be beneficial to allow users to upload as many images per entry as desired.

Are the conclusions about the tool and its performance adequately supported by the findings presented in the article?

Yes

Is the rationale for developing the new software tool clearly explained?

Yes

Is the description of the software tool technically sound?

Yes

Are sufficient details of the code, methods and analysis (if applicable) provided to allow replication of the software development and its use by others?

Partly

Is sufficient information provided to allow interpretation of the expected output datasets and any results generated using the tool?

Yes

Reviewer Expertise:

Automated image analysis in plant phenotyping

I confirm that I have read this submission and believe that I have an appropriate level of expertise to confirm that it is of an acceptable scientific standard, however I have significant reservations, as outlined above.

# Response

**Published:** 2022-09-14

**Authors:** Franco Röckel

**Affiliations:** Julius Kühn Institute (JKI) - Institute for Grapevine Breeding Geilweilerhof, Germany

First of all, we would like to thank the reviewer who gave us constructive comments and the opportunity to elaborate on certain aspects.To the review:

**"Dear authors,**

**This paper describes a potentially useful tool to address the issue of manual data collection from plant phenotyping experiments. Such a tool, if well executed, goes some way to providing for an information management system for collecting and recording phenotyping data in the field.**

**I have more experience with automated data collection though analysis of images, but I do recognise the need and utility of a tool to assist with data capture by hand.**

**The app seems to focus almost entirely on data collection, and as noted in a response to a previous review, this data can then be fed to further downstream processing outside the app as required. The app therefore seems to be a semi-constrained front end for entering data into an Excel/CSV format. Whilst a simple idea, I believe this is useful in practice. I have some comments below.**

**It is a shame that there is no enforcing of controlled vocabulary on some of the fields. In the previous response to authors it is noted that the use of internationally-recognised descriptors is recommended, so not having the option to enforce this in some scenarios feels like a missing feature which should be present."**

 We agree that a strict control of vocabulary and normed descriptors would be of outstanding importance. Nevertheless, the tool reflects the unsatisfactory situation that stakeholder, like funding agencies, publisher or international core database, implemented no mechanisms to ensure this. Rather, it is moved to the level of  informal commitments in institutional strategies. But even those need proper review. In order to provide a commonly usable data recording tool, the decision was to implement the data consistency in the database layer, which implements site-specific rules. On any inconsistency, a data steward is then responsible to resolve these. PhenoApp was built to be as much as possible customizable for maximum user friendliness. We do not want to enforce the use of any specific descriptors and consequently exclude users with individual (not yet harmonized) use cases. The controlled use of certain descriptors can easily be supervised on the institutional-wide level with read-only descriptor lists as explained in more detail in the next comment.

 However, we recognize the possible benefit of such a feature. The integration of a common set of descriptors for specific species with a standardized vocabulary could be implemented in future updates. Steps to facilitate species-level descriptor management are the basis for such an option and are already under development.

**"Related, a lot of emphasis is put on the input file, which I believe you use a lab information system to supply. How well practically do you see this working if the institute does not have a LIMS?"**

 At the beginning of the development, we did not use a LIMS either. We once created a common input file with a read-only descriptor list stored in an institutional-intern accessible folder. Therefore, users always used the same descriptor template and only added their specific plant list. It is more or less the same process as with a LIMS with a few more manual steps per user and without the automated LIMS database upload of the results after phenotyping. Since we use institutional-wide, standardized plant information (accession name, accession number, plant positions in the field etc.), the input files were completely standardized and the gathered information could be easily uploaded after the connection to our LIMS.

 To conclude, we already established the standardized, institutional-wide use before the implementation of a LIMS. This should be feasible for every institute/lab in comparable manners as described above.  

**"I agree with the previous reviewer raising questions about Excel as a choice of data format. I think there are issues with this, and perhaps more flexible formats like JSON should be explored in the future (as you mention). However, there is no doubt Excel provides accessibility and readability of data in a form most phenotyping researchers will be familiar with."**

 Indeed the use of JSON as standardized, well-defined file format would have simplified the development of PhenoApp and ensured a robust file un-marshalling and data structure consistency per-se. As argued before, one aim is to support a wide range of application areas and institutional infrastructures where some need to support Excel as widely used and accessible solution for data acquisition, editing and exchange. We saw the risk of JSON in losing those data maintainers and producers, who rely on proprietary historic database backend systems and data management processes. E.g. some do not use databases at all and archive in folder systems of Excel files as primary storage. 

**"The option of uploading images is welcome, especially as I can see the benefit in later using these alongside the manually collected data to support development of AI-based systems which can analyse the images. However, only allowing one image at a time seems like an oversight here. I am convinced it would be beneficial to allow users to upload as many images per entry as desired."**

 Please allow to clarfiy a potential misunderstanding. Only for a descriptor it is possible to upload one sample image. However, this should be sufficient to display representative images of rating schemes, phenological stages etc. in the app interface at the time of rating. Instead, for specific plant entries, it is not possible to upload images at all. However, we implemented the connection to the photo app of the devices used to take location-specific photos. There is no number limit for images per location here and naming is standardized based on the specified location, the actual timestamp and the variety name (if given) to ensure subsequent recognition. This allows clear allocation of the image to location/variety and if rating was performed for only one/few traits a clear allocation is possible even for the trait. Of course, these photos can be used for all downstream analysis without any restrictions.

# Peer review: Reviewer response for version 1

**DOI:** 10.5256/f1000research.77970.r119127

**Published:** 2022-01-25

**Authors:** Karin Köhl

**Affiliations:** 1 Max Planck Institute of Molecular Plant Physiology, Potsdam, Germany

Dear Authors,

 Your manuscript describes a very useful tool for manual phenotyping based on mobile devices. However, the information on the tool need some amendments, especially for a user who is not familiar with the Limsophy-DB and wants to use it independently. I have some experience with using mobile devices for high-throughput phenotyping as we designed a similar tool for WinCE in 2015 (see reference).

 Is the plant identification done by selection from a pre-existing list or by free text entry? In the latter case, how do you make sure the plant exists in the database/LIMS? How do you deal with duplication of the location names: two independent experiments may use the same combination of plot row and plant I.D? If the system is used independent of a LIMS system: How do you ensure that controlled vocabulary is used for species/variety? And, how do you ensure that controlled vocabulary for descriptors are used in the entry excel file.

 Using excel files as an entry format has risks. How do you avoid Excel reformatting numbers into dates? How do you avoid users messing up the file by accidentally including carriage returns and other control characters?

 For the numeric data entry: where do you define the unit in which the measurement was done (e.g. m, cm for height)?

 How many clicks do you need to enter the BBCH (Biologische Bundesanstalt für Land- und Forstwirtschaft, Bundessortenamt und CHemische Industrie) information? Can you just enter the code or do you have to select from a list? The latter is very time consuming when you have to score a large number of plants.

 How do you deal with multiple pictures of one BBCH stage, does the new image overwrite the old image or is it added?

 Can you store the specific selection of descriptors as a protocol for future use, to make sure that each time the same set of traits is phenotyped. Is it possible to store the protocol independent of the Limsophy-DB?

 Can you provide a reference for the Limsophy-DB?

 Output excel file: how do you record the meaning of Sb and Bl if the system is not linked to a LIMS system? How do you maintain the link between input and output file?

Are the conclusions about the tool and its performance adequately supported by the findings presented in the article?

Partly

Is the rationale for developing the new software tool clearly explained?

Yes

Is the description of the software tool technically sound?

Partly

Are sufficient details of the code, methods and analysis (if applicable) provided to allow replication of the software development and its use by others?

Partly

Is sufficient information provided to allow interpretation of the expected output datasets and any results generated using the tool?

Partly

Reviewer Expertise:

plant phenotyping, data management, abiotic stress tolerance

I confirm that I have read this submission and believe that I have an appropriate level of expertise to confirm that it is of an acceptable scientific standard, however I have significant reservations, as outlined above.

## References

[1] : A software tool for the input and management of phenotypic data using personal digital assistants and other mobile devices. *Plant Methods* .2015;11: 10.1186/s13007-015-0069-3 25 10.1186/s13007-015-0069-3 25866550PMC4393613

# Response

**Published:** 2022-02-23

**Authors:** Franco Röckel

**Affiliations:** Julius Kühn Institute (JKI) - Institute for Grapevine Breeding Geilweilerhof, Germany

First of all, we would like to thank the Reviewer who has provided us with many insightful comments and a chance to improve our work. To the review:

**Your manuscript describes a very useful tool for manual phenotyping based on mobile devices. However, the information on the tool need some amendments, especially for a user who is not familiar with the Limsophy-DB and wants to use it independently. I have some experience with using mobile devices for high-throughput phenotyping as we designed a similar tool for WinCE in 2015 (see reference).**

**Is the plant identification done by selection from a pre-existing list or by free text entry?** 

 The PhenoApp is designed for phenotyping of previously defined material with given locations (e.g. breeding material in field) that is screened with defined descriptors. This information is provided to the App in an Excel file as described under “Data import and export” prior to the phenotyping. In the ”Locations” sheet, the material can be filled with the individual material, while in the sheet “Traits” the descriptors can freely be customised. Data to the Excel Import file can be (1) entered directly, (2) by copy & paste from pre-existing lists/files, or (3) from databases (like LIMS) using matching data export settings.

**In the latter case, how do you make sure the plant exists in the database/LIMS?**

 There is no control via PhenoApp. If you use the PhenoApp independently of a database you can use the Excel output file with the data to feed every planned downstream application. For data import to a database/LIMS we highly recommend to use a script to create the input files and a second one that checks every entry during data import after phenotyping. This avoids errors *a priori* and gives full control over the clear allocation and completeness. 

**How do you deal with duplication of the location names: two independent experiments may use the same combination of plot row and plant I.D?**

 Each location (or plant) is linked to an experiment, so there are no problems if a location appears in several experiments. Thus, only the associated locations are used in each experiment. If a specific location is given multiple times in a single list with different names, all entries are displayed in PhenoApp, although only one is true and others are wrong. Users will recognize it and can then adapt the input file.

**If the system is used independent of a LIMS system: How do you ensure that controlled vocabulary is used for species/variety? And, how do you ensure that controlled vocabulary for descriptors are used in the entry excel file.**

 We designed PhenoApp to be as customisable as possible. There is no controlling of common vocabulary for any descriptor. Nevertheless, we recommend using international standardised descriptor lists as mentioned in the text. The validation of the correct taxonomy rely on a central defined vocabulary or ontology. This central place should be a database that offer access and validation features. Nevertheless, there are spreadsheet based terminology validation features, e.g. RightField (DOI: 10.1093/bioinformatics/btr312 ). Furthermore, one may even implement MS-Excel macros that validate to a remote accessible list of valid species.

**Using excel files as an entry format has risks. How do you avoid Excel reformatting numbers into dates?** 

 Indeed this is a potential pitfall as reported already in Browman et al. ( 10.1080/00031305.2017.1375989 ) By following these rules, there have been no problems of this kind with Excel so far. After phenotyping, dates were displayed correctly. During development, Excel seemed to be the simplest and most used format for scientists to define their experiments independently. In the future, it is planned to support JSON as an alternative input format. Furthermore, csv file format can be used for export instead of xlsx.

**How do you avoid users messing up the file by accidentally including carriage returns and other control characters?**

 So far, no problems of this kind were known and no controlling system is used. PhenoApp will display the special characters. In our institutes, the input files are mainly created directly by the LIMS to avoid these errors and where this is not possible, standardised input file templates are available. To a certain degree, users are responsible for the correctness of their own datasets.

**For the numeric data entry: where do you define the unit in which the measurement was done (e.g. m, cm for height)?**

 This can be specified in the descriptor itself. We recommend defining the measurement including the used unit as precisely as possible in the “Remark” field of the “Traits” sheet in the input file.

**How many clicks do you need to enter the BBCH (Biologische Bundesanstalt für Land- und Forstwirtschaft, Bundessortenamt und CHemische Industrie) information? Can you just enter the code or do you have to select from a list? The latter is very time consuming when you have to score a large number of plants.**

 Every pre-integrated BBCH scale has three list levels: species, macro stage and micro stage. Species and the macro stage need to be selected once in the beginning meaning that with every new plant entry both entries are kept. Only the micro stage needs obviously to be selected at every location entry. You need one click to open the micro stage menu and one additional click for the individual BBCH stage at every location. It is quite handy and approved. 

**How do you deal with multiple pictures of one BBCH stage, does the new image overwrite the old image or is it added?**

 There is only one image per stage/descriptor possible and a new image would replace the old one. If really two images are needed, users would have to merge them into one image file and then upload it. This has been done in the past. 

**Can you store the specific selection of descriptors as a protocol for future use, to make sure that each time the same set of traits is phenotyped. Is it possible to store the protocol independent of the Limsophy-DB?**

 We store our descriptor list in the LIMS database. This list is always used when an input file is created. Before we integrated the LIMS in the PhenoApp workflow, we created an empty input file (empty of plant locations) with a read-only descriptor list in Excel and saved it in an institutional-wide PhenoApp filesystem folder share for further use. Every new user was informed to rely on this standard list. Next to that, a user is free to adapt his individual descriptor list in the input file to only the relevant descriptors he is going to use in the given experiment or development state by deleting all others.

**Can you provide a reference for the Limsophy-DB?**

 Ghaffar, M., Schuler, D., Konig, P., Arend, D., Junker, A., Scholz, U., Lange, M., 2020. Programmatic Access to FAIRified Digital Plant Genetic Resources. J Integr Bioinform; DOI: 10.1515/ *jib*- *2019*- *0060*

**Output excel file: how do you record the meaning of Sb and Bl if the system is not linked to a LIMS system?** 

 A detailed meaning of every descriptor can be given in the remark column, which is a free text field. We recommend defining records of individual rating systems here as precisely as possible including units for measured values, phenological state, organ positions etc. 

**How do you maintain the link between input and output file?**

 The complete location list of the input file is also copied to your output file independent of your phenotyping.
